# 

**Published:** 1900-12-15

**Authors:** 


### NOTICE TO CORRESPONDENTS.

All MSS., Letters, Books for Review, and other matters intended for the Editor should be addressed The Editor, "The Hospital," The Hospital Building, 28 & 29 Southampton Street, Strand, W.C.
The Editor cannot undertake to return rejected MSS., even when accompanied by stamped directed envelope.

**Notice to Advertisers and Subscribers.**

All Advertisements, Orders for Copies of The Hospital, and Business Communications should be addressed to **THE MANAGER (Not to the Editor),** The Hospital, 28 & 29 Southampton Street, Strand, London, W.C.
The Cost of Subscription, post free, is as follows:—Per week, 2½d.; per quarter, 2s. 8d.; per half-year, 5s. 3d.; per annum, 10s. 6d. Foreign:—Per annum, 15s. 2d., payable, in all cases, in advance.

**NOTICE.—Vol. XXVIII. of "The Hospital" (April to September, 1900), in handsome cloth binding, is now on sale at the Office of this Journal, price, 7s. 6d. Cases for binding the Numbers contained in this Volume 1s. 6d. Index to Vol. XXVIII., 2½d., post free.**
**The Monthly Part for December is now ready. Price 6d., by post 8d.**

### BOOKS RECEIVED.

Vail and Co.

"Quarterly Report on the Health of Islington." By the Medical Officer of Health.

John Bale, Sons, and Danielsson.

"On the Treatment of Tuberculous Diseases in their Surgical Aspect. Being the Harveian Lectures for 1899." By W. Watson Cheyne, M.B.Edin., F.R.C.S.Eng., F.R.S.

"A Prescriber's Companion." By Thomas D. Savill, M.D.Lond., D.P.H.Camb. Third edition.

"Outlines for Dissectors." By F. G. Parsons, F.R.C.S.

Spottiswoode and Co.

"Indian and Colonial Addendum to the British Pharmacopœia, 1898."

Smith, Elder, and Co.

"On Some Cirrhoses of the Liver." By Walter Butler Cheadle, M.A., M.D.Cantab.

Charles Skipper and East.

"Report of the Medical Officer of Health for the Port of London."

The Brighton Railway Company.

"The Riviera and Italy for a £10 Note."

Baillière et Cie.

"Dictionnaire de Physiologie." Par Charles Richet.

"Premier Fascicule du Tome V."

Novello and Co.

"Seven Carols, including two War-Christmas Carols." Edited by Rev. Alfred A. Toms, M.A. Music by Rev. W. G. Whinfield, B.A., Mus.Bac. (Oxon.)

### Scraps and Gleanings.

The Princess of Wales has forwarded for the benefit of the inmates of the British Home and Hospital for Incurables at Streatham a most acceptable present of pheasants, partridges, hares, and rabbits, this charity being the first in this country to receive the patronage of Her Royal Highness.

A sum of £30,000 is required to enable the managers of the Glasgow Western Infirmary to commence the urgently-needed extensions and improvements.

The net amount produced by the bazaar in aid of the Victoria Central Hospital, Liscard, was about £5,300. The executive therefore will be relieved from immediate anxiety as to the finances, as only £4,000 is required to open the hospital.

The annual report of the Coventry and Warwickshire Hospital shows that the number of in-patients during the year had been 758, the largest number treated in one year in the history of the institution. The out-patients had numbered 4,995, as against 4,706 in the previous year.

This year's results of the Northampton Hospital Week Fund were £944 18s. 7d., and at the recent meeting of the committee the sum of £850 was voted to the Northampton Infirmary, thus leaving in hand £23 odd, after deducting the expenses, which amounted to £71.

The new building and consequent alteration to the older portions of the Croydon General Hospital, together with furnishing and equipment, have cost £11,258. Towards this amount £8,501 has been received, leaving a deficit of £2,757. As a result of these alterations there are now 94 beds available for patients.

The Local Government Board have been applied to by the Kent County Council for permission to borrow £5,700 for the purpose of erecting an isolation hospital for infectious diseases to serve the parishes of Chiddingstone, Cowden, Edenbridge, Hever, Penshurst, and Leigh.

The total receipts from the donations in connection with the Walsall Hospital Bazaar amounted to about £6,000, of which sum the takings on the three days represented £1,800. The object of this bazaar was to provide additional accommodation for the nurses, a new out-patients' department, and to wipe off the existing debt.

Dr. E. Magennis, J.P., 37 Harcourt Street, Dublin, has been the recipient of an address and presentation by the doctors resident in the Lurgan Union. The presentation consisted of a Harvard chair for use in his consulting room, and bore on it a silver plate with the following inscription: "Presented to Edward Magennis, Esq., M.D., J.P., by his fellow practitioners in and around Lurgan and Portadown on the occasion of his leaving for Dublin as a token of their good fellowship and esteem. September, 1900." The address was signed by every medical man in the district.

### The Student of Medicine.

### APPOINTMENTS.

**John Aitken,** M.B., Ch.B.Glas., appointed an Outdoor House Surgeon to the Maternity Hospital, Glasgow. **Stanley Boyd,** F.R.C.S., appointed Consulting Surgeon to the Paddington Green Children's Hospital. **Frederick F. Burghard,** F.R.C.S., appointed Surgeon to In-patients at the Paddington Green Children's Hospital. **Frank Butterfield,** M.B.Lond., appointed Senior House-Surgeon of the Blackburn and East Lancashire Infirmary. **John Henderson,** M.B., Ch.B.Glas., appointed an Outdoor House-Surgeon to the Maternity Hospital, Glasgow. **R. G. Hogarth,** F.R.C.S.Eng., appointed Surgeon to the Children's Hospital, Nottingham. **Robert G. Johnson,** M.R.C.S., L.R.C.P., appointed Registrar and Pathologist, North London Consumption Hospital. **J. Dickinson Leigh,** M.B., F.R.C.S.E., D.P.H., appointed Certifying Factory Surgeon for the Scarborough District. **George Millson,** L.R.C.P.Lond., M.R.C.S.Eng., appointed Medical Officer of Health for the Borough of Southwark. **John Wallace Milne,** M.B., C.M.Aberd., M.R.C.S.Eng., L.R.C.P.Lond., appointed Assistant-Surgeon in the Aberdeen Royal Infirmary. **James Scott,** M.A., M.B., Ch.B.Glas., appointed Indoor House-Surgeon to the Maternity Hospital, Glasgow. **W. R. Smith,** M.D.Lond., B.S., appointed Certifying Factory Surgeon for the Beeston District, co. Nottingham. **Ewen C. Stabb,** F.R.C.S., appointed Surgeon to Out-patients at the Paddington Green Children's Hospital. **W. A. Trumper,** L.R.C.S.Eng., L.R.C.P.Lond., appointed Assistant House-Surgeon to the Royal Devon and Exeter Hospital, Exeter. **Percy E. Turner,** M.B., B.S.Dunelm L.R.C.P., D.P.H.Oxon., appointed Superintendent of the Salvation Army Medical Department in India.

### THE HOSPITAL

### BOOKS OF REFERENCE ON HOSPITALS.

In Four Volumes, with a Portfolio of Plans. Royal 8vo. Top gilt. Cloth extra, bevelled.

Price of the Book, complete ... ... £8 8 0

Vols. I. and II. (only).—Asylums and Asylum Construction £4 10 0

Vols. III. and IV.—Hospitals and Hospital Construction, — with Portfolio of Plans £6 0 0

The Portfolio of Plans (20 ins. by 14 ins.) separately, price £3 3 0

"The collection of plans of hospitals will be invaluable to all architects and charitable committees intent upon building, and the whole book contains a vast collection of information not easily accessible in other treatise."
*Athenæum.*

"SIR HENRY BURDETT'S monumental work on Hospitals."
*The Times.*

### HOSPITALS AND ASYLUMS OF THE WORLD.

**Their Origin, History, Construction, Administration, Management, and Legislation.**

With Plans of the chief Medical Institutions, accurately drawn to a uniform scale, in addition to those of all the Hospitals of London in the Jubilee year of Queen Victoria's reign.

BY

**SIR HENRY BURDETT, K.C.B.**

*Only a few copies of this valuable work now remain unsold.*

"A book which should be found in all asylums, hospitals, and public libraries, and which every architect and medical man who aspires to an understanding of the principles of hospital construction, organisation, and management will do well to procure for himself."
*British Medical Journal.*

"The work is a monument of skilful labour in an admirable cause. . . . The largest and most valuable works in existence upon a subject of primary importance to the community."
*Morning Post.*

"A work which no architect taking up hospital construction as part of his professional work can afford to neglect."—*Builder.*

"Would be impossible to exaggerate the interest of the work."—*Saturday Review.*

"Such a mass of information was certainly never brought together before."
*Westminster Gazette.*

"This is the most extensive and elaborate work upon the subject of hospitals, including hospitals for the insane, which has ever been published in any language. We heartily commend the book to all those who take the slightest interest in our grand hospital system."—*Pall Mall Gazette.*

The SCIENTIFIC PRESS, LTD. 28 & 29 Southampton Street, Strand, London, W.C.

### Important Works on Hospitals & Allied Subjects.

BY
Sir HENRY BURDETT, K.C.B.
Third Edition.
Profusely Illustrated.
Crown 8vo.
Cloth gilt, 10/6.

### COTTAGE HOSPITALS:
### GENERAL, FEVER, AND CONVALESCENT.
Their Progress, Management, and Work in Great Britain, Ireland and the United States.

"A storehouse of practical information in which the founders of a new hospital or the managers of an old one may find trustworthy guidance with regard to nearly everything which they should either do or avoid."
*The Times.*

BY
Sir HENRY BURDETT, K.C.B.
Published Annually.
Crown 8vo., Over 1,000 pp.
Cloth gilt, 5/-

### BURDETT'S HOSPITALS & CHARITIES.
THE YEAR BOOK OF PHILANTHROPY
AND
HOSPITAL ANNUAL.

"We welcome each reappearance of this annual, whose worth is so well known that it has become indispensable to those engaged in the management of old hospitals or construction of new ones."
*British Medical Journal.*

"The almost indispensable companion of all who are in any way connected with charity."
*Saturday Review.*

"There is no other book that covers the whole ground this volume does."—*Spectator.*

BY
Sir HENRY BURDETT, K.C.B.
Published Annually.
Crown 8vo.
Cloth gilt, 5/-

### BURDETT'S OFFICIAL NURSING DIRECTORY.

"Has no equal as a guide and book of reference for a large and increasing class of philanthropic and professional workers."
*Daily Chronicle.*

"Of great usefulness to the medical and nursing profession as well as the public generally."
*St. James's Gazette.*

BY
Sir HENRY BURDETT, K.C.B.
Published Annually.
Crown 8vo. Over 300 pp.
Cloth, 2/- net;
post free, 2/4.

### THE NURSING PROFESSION:
### HOW AND WHERE TO TRAIN.
Being a Guide to Training for the Calling of a Nurse.

"The book is well compiled."
*Lancet.*

"Brings the whole nursing field under review. Would-be nurses will find all the information they need in deciding upon the Institutions at which to go through their training and find employment afterwards."—*Graphic.*

"A more complete professional book it would be hard to imagine, and perhaps impossible to discover."—*Churchwoman.*

Reprinted
from
"THE HOSPITAL."
Crown 8vo.
Cloth, 2/6.

### HOSPITAL EXPENDITURE:
### THE COMMISSARIAT.
Every Institution should preserve this book at hand for constant reference.

"Should prove a valuable handbook to those upon whom rests the responsibility of hospital finance, and should be of interest to the public as giving a fair idea of what this department should and does cost."—*Manchester Courier.*

"A thoughtful, well-informed, and particularly detailed study of the finance that regulates the victualling department of a large hospital. The well-digested data of the book, and its careful setting forth of sound principles of domestic economy cannot but prove serviceable to hospital managers in the ordering of their affairs."
*Scotsman.*

The SCIENTIFIC PRESS, LTD., 28 & 29 Southampton Street, Strand, London, W.C.

### A LIBRARY OF

### POPULAR TEXT-BOOKS ON NURSING.

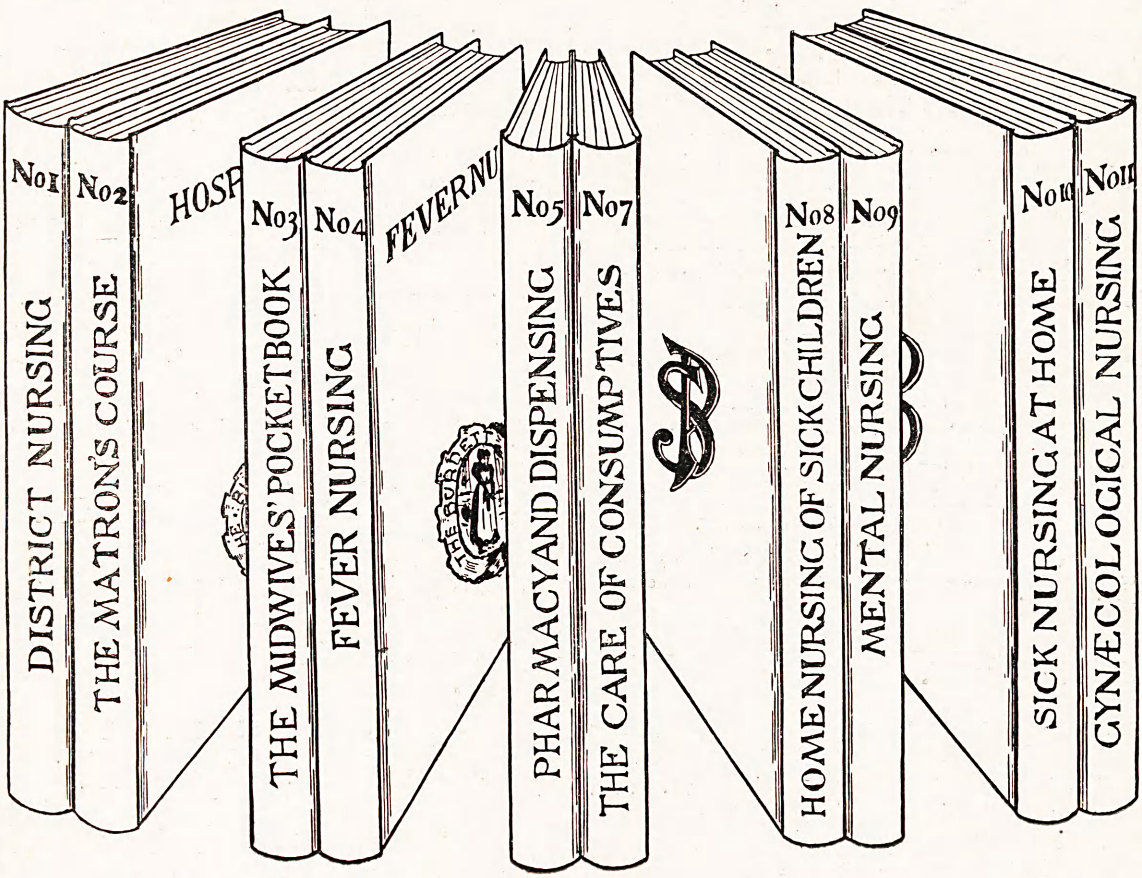

### "THE BURDETT SERIES."

Each Volume contains about 100 pages written by a competent authority on the subject treated.

**Actual Size $6\frac{3}{4}$ in. by $4\frac{1}{4}$ in. Bound in Cloth. Price 1s. each, post free.**

No. 1.—**Practical Hints on District Nursing.** By Miss AMY HUGHES.

No. 2.—**The Matron's Course.** An Introduction to Hospital and Private Nursing. By Miss S. E. ORME.

No. 3.—**The Midwives' Pocket Book.** By Miss HONNOR MORTEN, Author of "The Nurse's Dictionary," &c., &c.

No. 4.—**Fevers and Infectious Diseases:** their Nursing and Practical Management. By WILLIAM HARDING, M.D.Ed., M.R.C.P.Lond.

No. 5.—**Notes on Pharmacy and Dispensing for Nurses.** By C. J. S. THOMPSON.

No. 7.—**The Care of Consumptives.** By W. H. DAW, M.R.C.S., L.R.C.P.Lond.

No. 8.—**Home Nursing of Sick Children.** By J. D. E. MORTIMER, M.B., F.R.C.S., L.S.A.

No. 9.—**Mental Nursing.** By WILLIAM HARDING, M.D.Ed., M.R.C.P.Lond.

No. 10.—**Sick Nursing at Home.** By Miss L. G. MOBERLY.

No. 11.—**Gynæcological Nursing.** By G. A. HAWKINS-AMBLER, F.R.C.S.

---

*TO BE OBTAINED OF ALL BOOKSELLERS, OR OF*

The SCIENTIFIC PRESS, LTD., 28 & 29 Southampton Street, Strand, London, W.C.

### STANDARD NURSING TEXT-BOOKS.

### A Handbook for Nurses. By J. K. WATSON, M.D., M.B., C.M.

Crown 8vo, profusely illustrated with over 60 Cuts, cloth gilt, 5s.

This Handbook is an entirely new departure in Nursing literature, inasmuch as it contains useful information on Medical and Surgical matters hitherto only to be obtained from expensive works written expressly for medical men.

"A concise and comprehensive exposition of as much medical knowledge as a nurse needs. The work cannot but prove useful for whom it has been designed."—*Scotsman.*

### 3s. 6d. Series.

### Nursing: its Theory and Practice.

By PERCY G. LEWIS, M.D., M.R.C.S., L.S.A.

New Edition. Crown 8vo. Illustrated, cloth.

"Nurses will find the book full of interest and instruction, which cannot fail to assist them in their work."—*British Medical Journal.*

### Surgical Ward-work and Nursing.

By ALEXANDER MILES, M.D.Edin., C.M., F.R.C.S.E.

Revised Edition. Demy 8vo. Illustrated, cloth gilt, 3s. 6d. net.

"An illustrated manual which will furnish much needed guidance to the student and the nurse in the early stages of their apprenticeship."
*The Times.*

### First Aid to the Injured and Management of the Sick.

By E. J. LAWLESS, M.D., D.P.H.

New Edition. Crown 8vo. Illustrated, cloth gilt.

"Those engaged in instructing classes in administering first aid will find this book invaluable."—*Pall Mall Gazette.*

### Ophthalmic Nursing.

By SYDNEY STEPHENSON, M.B., F.R.C.S.E.

Crown 8vo. 200 pp. Illustrated, cloth.

"May very advantageously be studied by nurses and clinical clerks or dressers who are about to enter the Ophthalmic service of a hospital."—*Lancet.*

### 2s. 6d. Series.

### Elementary Anatomy and Surgery for Nurses.

By WILLIAM MCADAM ECCLES, M.S.Lond., M.B., &c.

Crown 8vo. Illustrated, cloth.

"Valuable to nurses who are beginning the study of anatomy. The instruction is given very clearly and concisely, and the reader is able to glean much information in a very condensed form."—*Nursing Notes.*

### Nursing in Diseases of the Throat, Nose, and Ear.

By P. MACLEOD YEARSLEY, F.R.C.S.Eng., M.R.C.S., L.R.C.P.Lond.

Demy 8vo. Illustrated, cloth.

"Of practical service to every nurse who wishes to be thoroughly trained."—*Scottish Medical and Surgical Journal.*

### Hospital Sisters and their Duties.

By EVA C. E. LÜCKES, Matron to the London Hospital.

Third Edition. Crown 8vo. Cloth gilt.

"An admirable guide to the beginner anxious to take up a Sister's work. Its pages bring help and knowledge imparted in a very sympathetic, straightforward manner by one who has had practical experience in the lesson she teaches."—*Gentlewoman.*

### The Nurse's Dictionary of Medical Terms and Nursing Treatment.

Compiled by Miss HONNOR MORTEN.

Revised Edition. Demy 16mo. Cloth, 2s.; leather, 2s. 6d. net.

"A very useful little book for reference, and should be at the disposal of every nurse."—*Birmingham Medical Review.*

### USEFUL BOOKS RELATING TO:—

### CHILDREN.

### Spinal Curvature and Awkward Deportment: their Causes and Prevention in Children.

By GEORGE MÜLLER.

English Edition, edited and adapted by RICHARD GREENE, F.R.C.P.Ed.

Crown 8vo. Illustrated, cloth gilt, 2s. 6d.

"Gives directions which any intelligent parent or teacher could carry out with the help of very simple apparatus, showing how to avoid, or, in early cases, to cure, certain malformations, which, in the majority of cases, arise either from the neglect of very simple hygienic rules or from carelessness."—*Daily Chronicle.*

### The Mother's Help and Guide to the Domestic Management of her Children.

By P. MURRAY BRAIDWOOD, M.D.

Second Edition, with Addenda. Crown 8vo. cloth, 2s.

"We can confidently recommend the book for the purpose for which it is intended, and it contains many hints that would be valuable to the junior practitioner."—*Bristol Medico-Chirurgical Journal.*

### The Physiological Nursery Chart.

Designed by ERIC PRITCHARD, M.A., M.D.Oxon.

Price 1s. Post free 1s. 3d.

This chart has been designed for the purpose of registering the weight of an infant from birth. Full directions are given for preparing the food required by a normal child at various ages. Many useful "nursery aphorisms" are also appended.

### HYGIENE.

### Helps in Sickness and to Health: Where To Go and What To Do.

By Sir HENRY BURDETT, K.C.B.

Crown 8vo., 400 pp. Illustrated, cloth gilt, 5s.

"An excellent compendium . . . . Owing to the mass of facts and useful information which it contains on every topic in connection with the subject of health in the household, it should find a place in every layman's library."—*Medical Press and Circular.*

### A Manual of Hygiene for Nurses.

By JOHN GLAISHER, M.D., D.P.H.

Crown 8vo. Illustrated, cloth, 3s. 6d.

"So much vital knowledge in so convenient a form makes this handbook very valuable. It will prove an excellent adviser to nurses, and would by no means be out of place in the hands of parents and householders."—*Aberdeen Free Press.*

### DIET.

### Art of Feeding the Invalid.

By a Medical Practitioner and a Lady Professor of Cookery.

New and Popular Edition. Demy 8vo. cloth, 1s. 6d.

"A new edition of this valuable work is very welcome, and we heartily commend it to all who desire reliable and practical instruction in sick cookery."—*Nursing Notes.*

### The Physiological Feeding of Infants.

By ERIC PRITCHARD, M.A., M.D.Oxon.

Demy 8vo. Paper covers, 1s.

"The way the subject is dealt with is admirable, and the intelligent use of the book and the chart is likely to save many an infant's life."—*The Hospital.*

### Infant Feeding by Artificial Means.

A Scientific and Practical Treatise on the Dietetics of Infancy.

By Mrs. S. H. SADLER.

Second Edition. Crown 8vo. cloth gilt, 5s.

"Mrs. Sadler's book contains a very useful collection of the views of the best-known English authorities upon the subject."—*Daily Chronicle.*

### Diet in Sickness and in Health.

By Mrs. ERNEST HART.

Demy 8vo. Illustrated, blue buckram, gilt, 3s. 6d.

"We have perused this book with great pleasure, and feel sure that many will find in it much to lighten the days of those whose digestion is enfeebled."—*The Hospital.*

### MASSAGE.

### Art of Massage.

By A. CREIGHTON HALE.

Demy 8vo. Profusely illustrated, cloth gilt, 6s.

"Mrs. Hale seems to have had considerable experience, not only as a practitioner, but as a teacher of the art, and explains in a series of clearly illustrated articles the many complaints for which massage has been found beneficial, and the special manipulations required in each case."—*Morning Post.*

The SCIENTIFIC PRESS, LTD., 28 & 29 Southampton Street, Strand, London, W.C.

Printed by SPOTTISWOODE & CO., New Street Square, E.C.; and Published by the Proprietors, THE SCIENTIFIC PRESS LIMITED, at 28 & 29 Southampton Street, Strand, London, W.C.

